# The Dark Side of Organizational Identification: A Multi-Study Investigation of Negative Outcomes

**DOI:** 10.3389/fpsyg.2020.572478

**Published:** 2020-09-29

**Authors:** Muhammad Irshad, Sajid Bashir

**Affiliations:** Department of Management Sciences, Capital University of Science and Technology, Islamabad, Pakistan

**Keywords:** organizational identification, psychological entitlement, unethical pro-organizational behavior, pro-social rule-breaking, externally motivated organizational citizenship behavior, leader–member exchange

## Abstract

After more than two decades of research on the positive side of organizational identification, researchers have begun to realize that it also has a dark side that needs immediate consideration. With support from social identity theory, the current study sheds light on the understudied role of the dark side of organizational identification by investigating its indirect effects on (a) psychological entitlement, (b) unethical pro-organizational behavior, and (c) pro-social rule-breaking through externally motivated organizational citizenship behavior, taking leader–member exchange as a boundary condition. Two surveys were conducted to test the proposed moderated mediation model. Data for the study 1 was collected from employees (*N* = 356) working in the service sector (i.e., Universities, Banks and Telecommunication Organizations), whereas responses for study 2 were taken from employees (*N* = 259) working in the hospitality industry. A time-lagged research design was selected for both surveys to avoid common method bias. The results demonstrate that organizational identification leads to adverse outcomes in the form of psychological entitlement, pro-social rule-breaking and unethical pro-organizational behavior through externally motivated organizational citizenship behavior. Furthermore, a high-quality leader–member exchange relationship enhances these indirect effects of organizational identification. Several theoretical and practical implications, along with limitations and future research directions, are also discussed.

## Introduction

Since its inception, organizational identification (OI) has been considered a source of positive employee outcomes (Tarakci et al., 2018). Most definitions also suggest that it represents something positive. For example, Mael and Ashforth (1992) define it as “perceived oneness with the organization.” However, researchers have begun to realize that OI might not be as beneficial for organizations as it seems, mainly because it might cultivate negative emotions and behaviors among employees (Naseer et al., 2019). This calls for further investigation of the dark side of organizational identification, as repeatedly called for by different organizational behavior researchers (for references, see Galvin et al., 2015; Ashforth, 2016; Conroy et al., 2017).

The literature on the dark side of OI is still in its infancy stage some studies have highlighted its adverse outcomes such as UPOB (Umphress et al., 2010; Effelsberg and Solga, 2015), reduced cooperation (Polzer, 2004), work-family conflict (Li et al., 2015), resistance to change (van Dijk and van Dick, 2009), and psychological entitlement (Naseer et al., 2019). All these studies are either organization directed or self-directed adverse outcomes. Our study has focused on the dark side of OI from multiple perspectives, i.e., self-oriented (PE), organization-oriented (UPOB), and organizational stakeholder-oriented (PSRB). The existing literature on the dark side of OI suggests that the negative outcomes of OI are subject to underlying mechanisms and boundary conditions, which still need to be tested to fully understand the factors that cause employees to exhibit negative attitudes and behaviors as a result of OI (Ashforth, 2016; Chen et al., 2016; Naseer et al., 2019). In light of these limitations and inconsistencies, the current study aims to test an underlying mechanism and boundary condition through which OI leads to adverse outcomes like self-serving, deviant, and unethical employee behaviors (Mackey et al., 2019).

For decades, OI has been acknowledged to enhance organizational citizenship behavior among employees, but what if employees display citizenship behavior due to external or controlled motivation to constantly benefit the organization? Externally motivated organizational citizenship behavior (EMOCB) has recently emerged as a negative side of OCB. It is based on the notion that employees do not engage in OCB out of free will; instead, they are expected by citizenship norms to display extra-role behaviors, leaving them with no other option than to do much more than their formal job description (Yam et al., 2017). Since employees with higher levels of OI feel obligated to fulfill organizational norms due to a feeling of togetherness and shared goals, they are more likely to display a certain amount of EMOCB over and above internally motivated OCB (IMOCB).

The limited research on EMOCB suggests that it does more harm than good to the organization. EMOCB involves all extra-role behaviors that are not part of an employee’s job description (Yam et al., 2017). Researchers have highlighted several negative outcomes associated with it, such as workplace deviance (Yam et al., 2017). Engagement in externally motivated extra-role behavior can lead employees to a state of psychological entitlement (PE), in which they think they are worthy of praise and have earned the right to special treatment by performing extra tasks beyond their formal job duties (Harvey and Harris, 2010; Cooper et al., 2018).

Nonetheless, employees with high OI do not stop here, they further extend EMOCB by engaging in pro-organizational and pro-social behavior even when unethical and against organizational rules (Ryan and Deci, 2000; Chen et al., 2016; Yam et al., 2017; Naseer et al., 2019). This is mainly because of their loyalty and attachment to the organization, which motivates them to engage in pro-organizational behaviors even at others’ expense (Ashforth et al., 2008). Two pro-organizational behaviors currently receiving attention are unethical pro-organizational behavior (UPOB), which refers to behaviors that are in the better interest of the organization but are unethical (Umphress and Bingham, 2011), and pro-social rule-breaking (PSRB), which is defined as the violation of organizational rules in the better interest of organizational stakeholders (Brief and Motowidlo, 1986). When employees high in OI engage in OCB not because they want to but because they believe they have to, they might end up displaying UPOB and PSRB as a way of showing the organization that they are willing to go out of their way to ensure the organization’s success.

OI researchers have also considered boundary conditions in the relationship between OI and its negative consequences, as various personality dispositions, interpersonal factors, and situational variables might shape the relationship between OI and employee outcomes (Collins et al., 2019; Naseer et al., 2019). One such potential boundary condition that has received little attention is leader–member exchange (LMX). Leader–member exchange has mostly been examined as a predictor of OI (Katrinli et al., 2008; Loi et al., 2014). However, recent studies have identified it as an essential moderator between OI and employee outcomes and have called for further research on its role as a boundary condition (Liu et al., 2011; Zhao et al., 2019; Teng et al., 2020). Matta and Van Dyne (2015) have called for exploring the adverse outcomes of LMX in certain conditions. They suggest that contrary to popular belief, in-group members do not simply enjoy the benefits of having a high-quality relationship with their leader; they also have to do something extra to meet their leader’s expectations. Such expectations lead to extra-role performance and EMOCB, which is not necessarily based on employees’ autonomous motivation. Hence, we suggest that in-group members high in OI engage in both EMOCB and IMOCB to meet the double criteria of OI and LMX. In light of this, the current study also tests a boundary condition of the OI-outcomes relationship, namely LMX, which is a relatively understudied situational factor for adverse outcomes.

To summarize, the current study investigates the indirect effect of OI on (a) psychological entitlement, (b) UPOB, and (c) PSRB through externally motivated OCB. The current study further examines LMX as a boundary condition between OI and EMOCB. The selection and placement of variables in the proposed model is based on social identity theory (Tajfel and Turner, 1985). This theory states that people choose to join those groups that have higher status and similar values. Congruence in values develops identification, which motivates people to associate themselves with powerful people in the group. In every group, members not only enjoy the benefits of association, they also have to follow certain group norms (Jones and Volpe, 2011). When people fulfill their expected role, it enhances their self-esteem and encourages them to stand up for the group by engaging in activities that are beneficial to the group as a way of enhancing their status (Scott, 2007). Employees who strongly identify with their organization thus feel bound to fulfill their organization’s expectations by engaging in EMOCB. Employees high in OI who are additionally part of the leader’s in-group could feel more burdens of expectations to engage in EMOCB. Engagement in EMOCB enhances employees’ self-esteem and motivates them to engage in UPOB and PSRB.

In a causal study, it is important to ensure that all variables are different from each other and the relationships are in the right direction. We believe that all study variables are different from each other, with the exception of minor overlaps. For instance, organizational identification and leader–member exchange share some characteristics like strong bonding and affiliation, but these feelings are directed toward different sources. OI is directed toward the organization and reflects an employee’s closeness with his/her organization (Tarakci et al., 2018), whereas LMX refers to the exchange relationship between the leader and his/her followers (Loi et al., 2014). Furthermore, all three dependent variables, i.e., PE, UPOB, and PSRB exhibit visible differences in terms of their characteristics. PE is self-serving behavior in which employees think they deserve better treatment (Cooper et al., 2018), UPOB is unethical behavior that benefits the organization (Umphress and Bingham, 2011) and PSRB is a social variable encompasses rule-breaking for social purposes, such as for the benefit of colleagues, customers or other stakeholders, but at the cost of organizational rules (Ghosh and Shum, 2019). Hence, the current study focuses on investigating the antecedents of self-serving behavior (PE), pro-organizational behavior (UPOB), and pro-social behavior (PSRB).

## Theory and Hypothesis Development

### Overarching Theory

**FIGURE 1:**
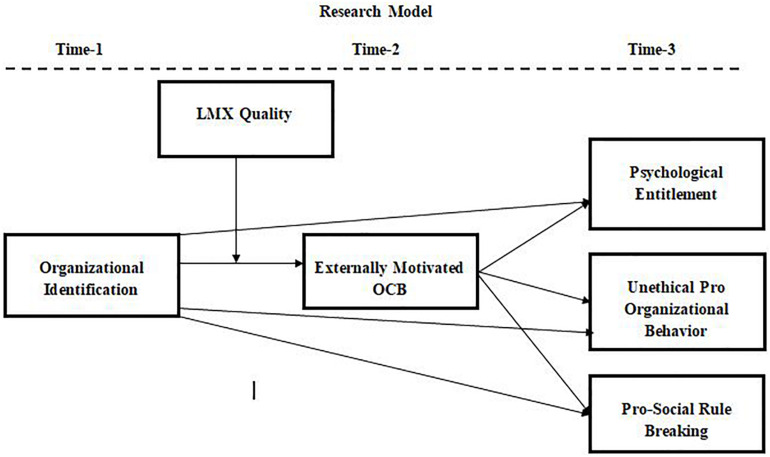
Proposed model diagram for study 1 and study 2 in time lags.

This study explains how and when OI leads to destructive outcomes by relying on social identity theory (Tajfel and Turner, 1985). Considering the tenants of social identity theory, we believe that organizations act as a group, and employees are the members of this group. Employees are obligated to do what is expected of them in order to maintain their jobs. Since organizations these days expect their employees to engage in OCB (Yam et al., 2017), employees high in OI engage in EMOCB in order to maintain their status. According to social identity theory, identity with any group enhances self-esteem and gives members the courage to go to extra mile to benefit the group (Tajfel and Turner, 1985). The self-esteem of employees high in OI increases after engaging in EMOCB due to their inner belief that they have done what is expected of them, which is why employees high in OI should tend to feel psychologically entitled. Similarly, employees high in OI do not hesitate to engage in PSRB and UPOB to benefit their organization. It is their way of showing the organization that they value their membership and are willing to do anything to maintain the organization’s superiority. The more strongly an individual identifies with the group, the more pro-organizational activities he/she engages in. Since employees who are part of the leader’s in-group strongly identify with the organization due to their bonding with both the organization and the leader, they engage in EMOCB to satisfy the leader as well as the group. Figure 1 shows the proposed theoretical framework for study 1 and study 2.

### Organizational Identification and Psychological Entitlement

OI refers to perceived oneness and high congruence between employees’ values with those of their organization (Tarakci et al., 2018). OI facilitates individual in developing connections in the power circles within the organization, and thus, one becomes a part of close organizational community/environment in which important information are openly shared, significant decisions are discussed and organizational strategies are debated (Avanzi et al., 2015). The existing studies also suggest that highly identified employees receive organizational support due to their closeness with the organization (Avanzi et al., 2018). Highly identified employees share a special bond with their employing organization which is based on common goals, norms and values. This bond distinguishes them from others by enabling them to enjoy privileges based on their closeness with the organization (Liu et al., 2011; Avanzi et al., 2015). This privilege, which is contingent upon the display of OI augments psychological entitlement in the individual (Tufan and Wendt, 2019). Psychologically entitled employees expect preferential treatment from the organization irrespective of their efforts (Żemojtel-Piotrowska et al., 2015). This expectation of special treatment is the result of access to resources, strong affiliation with the power figures and high bonding with the organization (Galvin et al., 2015; Naseer et al., 2019). Highly identified employees view themselves as important members of the organization to the extent that they start to believe that the organization cannot work properly in their absence (Galvin et al., 2015). Greater control over the organizational matters which is the result of OI gives employees the feeling that they are important for organizational success which develops feeling of entitlement (Chatterjee and Hambrick, 2007; Naseer et al., 2019). Social identity theory (Tajfel and Turner, 1985) also supports the association between organizational identification and psychological entitlement. According to this theory, employees become members of those groups whose values are aligned with their own, and this membership and assimilation in turn enhance self-esteem, pride, and feelings of entitlement (Naseer et al., 2019). Hence, we propose the following hypothesis in both study 1 and 2:

H1: OI is positively associated with employees’ PE.

### Organizational Identification and Unethical Pro-organizational Behavior

Organizational members high in OI share personal stakes with their organization and consider the organization’s success as their own (Mael and Ashforth, 1992). These employees’ sole objective is to promote their organization, even if it requires them to engage in unethical behaviors (Blader et al., 2017). One form of unethical behavior gaining attention these days is UPOB due to organizations’ direct or indirect involvement in promoting these behaviors (Chen et al., 2016). Employees high in OI frequently engage in unethical pro-organizational behavior in order to promote their organization at the cost of all other stakeholders (Umphress and Bingham, 2011). Highly identified employees disregard moral standards and do not hesitate to engage in UPOB to maintain their positive self-image, which is directly associated with the organizational image (Martin et al., 2014). Zuber (2015) is of the view that highly identified employees considered threats directed toward the organization as a direct threat to their identity, which motivates them to do everything they can to protect their shared self-image. Highly identified employees are willing to bypass ethical standards for the sake of the organization (Ashforth and Anand, 2003), which is why they are flexible to display unethical pro-organizational behaviors. Highly identified employees rationalize unethical behaviors by considering them crucial for protecting the shared self-image, as their ultimate objective is to benefit the organization (Campbell and Göritz, 2014). Some researchers believe that highly identified employees engage in unethical pro-organizational behavior to show their higher level of belongingness with the organization (Leavitt and Sluss, 2015). Ploeger and Bisel (2013) suggest that OI promotes in-group biases as a result of which employees engage in unethical behaviors that benefit their organization. Social identity theory also supports this association by stating that members of a particular group engage in activities that promote their group’s chances of success over others, because their group’s success also enhances their self-worth due to shared identity (Tajfel and Turner, 1985). Therefore, we hypothesize following in study 2:

H2: OI is positively associated with employees’ UPOB.

### Organizational Identification and Pro-social Rule-Breaking

Highly identified employees want what is best for their organization even if requires them to do more than their formal responsibilities as their self-identity is linked with the organization (Brown, 2017). The loyalty with the organizations motivates them to look for different means for profiting their organization and its key stakeholders. PSRB is also intended to benefit the organization and other organizational stakeholders. Unlike other employees, highly identified employees receive social support from their employing organization, which encourages them to engage in PSRB as they know that their organization is at their back (Avanzi et al., 2015, 2018). OI promote organizational commitment, which encourages employees to break the rules if it benefits the organization (Dávila, 2012). The ultimate objective of highly identified employees is to do what is best for the organization and its stakeholders. The existing research also suggests that highly identified employees raise their voice for the betterment of the organization (Qi and Ming-Xia, 2014; Wang et al., 2018). Identified individuals consider organizational harm as personal harm, and they are willing to deviate from rules to protect their organization (Zuber, 2015). They often engage in those behaviors which benefit the organization, such as PSRB, as the organizational benefit is linked to their benefit. Identified employees experience in-group biases, which encourage them to engage in unacceptable behaviors such as rule breaking as long as those actions benefit the organization (Ploeger and Bisel, 2013). To summarize, OI enhances loyalty and organizational commitment among employees, which motivates them to engage in all those behaviors which offer benefit to the organization even at the cost of compromising the rules. Social identity theory supports this notion by suggesting that members of a specific group will do anything they can to benefit their group. Hence, we propose the following hypothesis in study 2:

H3: OI is positively associated with employees’ PSRB.

### Mediating Role of Externally Motivated Organizational Citizenship Behavior Between Organizational Identification and Psychological Entitlement

Due to shared values, employees high in OI consider it their responsibility to follow the organization’s norms and engage in all behaviors that are beneficial for the organization (Tajfel and Turner, 1986; Ye, 2012). One well-known form of behavior that benefits the organization is OCB (Podsakoff et al., 2018). Contrary to the popular notion that OCB is voluntary behavior, researchers have started to observe that OCB has become a “must-do” activity in contemporary organizations (Ocampo et al., 2018). Several studies have found that modern organizations encourage their employees to engage in OCB in order to lubricate the organizational machinery for effective functioning (Bolino et al., 2013, 2015). In other words, employees are expected to participate in OCB to maintain their jobs (Yam et al., 2017). As this type of OCB is displayed due to external/controlled motivation rather autonomous motivation, it is termed as EMOCB (Yam et al., 2017).

Individuals higher in OI are more prone to engage in EMOCB, as they are willing to do anything in line with the organization’s expectations (Newman et al., 2016). Existing research further indicates that EMOCB not only benefits the organization, it also enhances employees’ expectations of their organizations (Newman et al., 2016; Yam et al., 2017; Langerud and Jordan, 2018). Yam et al. (2017) conducted an interesting study directing attention to this very association by proving that EMOCB acts as a perfect soil for cultivating a feeling of PE among employees. The external motivation to display OCB gives rise to a feeling of entitlement among employees, in which they start to think that they deserve special treatment from their organization as a result of the extra time and energy they were advised to invest in extra-role behaviors (Alkahtani, 2015). Seeking preferential treatment is a notable characteristic of psychologically entitled employees (Żemojtel-Piotrowska et al., 2015). Yam et al. (2017) also state that a state of psychological entitlement arises when employees are motivated to go above and beyond their formal work requirements by their employing organization due to their association. Hence, we propose the following hypothesis in both study 1 and 2:

H4: EMOCB mediates the relationship between OI and PE.

### Mediating Role of Externally Motivated Organizational Citizenship Behavior Between Organizational Identification and Unethical Pro-organizational Behavior

Chen et al. (2016) found that OI is a strong predictor of UPOB. However, this relation is subject to some underlying mechanisms (Blader et al., 2017). OI integrates employees’ inner self with organizational membership, which obligates them to work for the organization’s better interest. Nowadays, citizenship behaviors are expected of employees (Bolino et al., 2018). As members of the group, the primary focus of employees high in OI is to meet and exceed these expectations regarding citizenship behavior (Chen et al., 2016). Organizational citizenship norms encourage employees high in OI to work for the betterment of the organization as a result of their group membership (Cornwell et al., 2018), which leads them to engage in EMOCB. When employees feel that organizational norms demand OCB, they seek to exhibit all those behaviors that are beneficial for the organization without taking into account moral and ethical values (Bolino and Klotz, 2015). Researchers believe that the expectation to display OCB leads to deviant and unethical behavior at the workplace (Bolino and Klotz, 2015). Umphress et al. (2010) are of the view that unethical behaviors can be pro-organizational in nature. The existing literature suggests that when organizational norms encourage employees to perform extra-role behaviors, employees expand these citizenship behaviors to include UPOB in order to promote organizational functioning (Liu et al., 2019). Hence, we propose following hypothesis in study 2:

H5: EMOCB mediates the relationship between employees’ OI and UPOB.

### Mediating Role of Externally Motivated Organizational Citizenship Behavior Between Organizational Identification and Pro-social Rule Breaking

Bolino et al. (2013) term EMOCB as the dark side of OCB. When OCB is ingrained as an important activity in the organizational culture, employees extend their citizenship behavior to include rule-breaking in order to perform one’s job activities efficiently and extend one’s support of customers and coworkers (Bolino et al., 2018; Koopman et al., 2019). As previously stated, OI increases motivation among employees to engage in EMOCB, and employees expand their EMOCB to include deviant behavior in the form of PSRB. Borry (2017) highlighted that organizational norms are the gateway to rule-bending and rule-breaking behaviors. Rules compliance is dependent on endorsement by management (Fleming, 2019). When EMOCB becomes an organizational requirement, it leads to breaking organizational rules for pro-organizational purposes (Liu et al., 2019). Thus, it is proposed that external motivation to display citizenship behavior leads to PSRB, because employees deem such behavior an extension of OCB that contributes to the organization’s success. Social identity theory also supports this notion that employees unconditionally extend their work to support the group to which they belong and would do anything that is beneficial for the group. Since employees with higher levels of OI are obligated to fulfill organizational norms due to their in-group assimilation, they are more likely to display EMOCB due to group expectations (Tajfel and Turner, 1985). Hence, we propose following hypothesis in study 2:

H6: EMOCB mediates the relationship between employees’ OI and PSRB.

### Moderating Role of Leader–Member Exchange

High-quality LMX promotes extra-role behavior among employees due to the fostering of a good relationship with the supervisor (Bowler et al., 2019). High-quality LMX creates implicit pressure for employees to engage in citizenship behavior (Farmer et al., 2015). Huang et al. (2014) state that LMX quality and OI are potential predictors of citizenship behavior. In light of the existing literature, the current suggests that employees who have a high-quality relationship with their leader are more likely to display EMOCB as a result of OI. This is mainly because individuals high in OI who are also part of the leader’s in-group have two reasons to exhibit positive behavior: their shared identity with the organization and their close relationship with the leader (Tajfel and Turner, 1985). Both of these external sources of motivation promote EMOCB. Hence, we propose following hypotheses in both study 1 and 2:

H7: LMX moderates the relationship between OI and EMOCB, such that this relationship will be stronger when the quality of LMX is high and weaker when the quality of LMX is low.

The current study further proposes that leader–member exchange conditionally affects the indirect impact of OI on PE, UPOB and PSRB through EMOCB. We believe that employees who have a high-quality LMX relationship with their leader are more likely to engage in EMOCB, which gives them a sense of pride and PE, UPOB and PSRB compared to employees with a low-quality relationship. Hence, we hypothesize the following in both study 1 and 2:

H8: LMX moderates the positive indirect effects of OI on employee PE, such that the indirect effect through EMOCB is stronger when LMX quality is high and weaker when LMX quality is low.

H9: LMX moderates the positive indirect effects of OI on employee UPOB, such that the indirect effect through EMOCB is stronger when LMX quality is high and weaker when LMX quality is low.

H10: LMX moderates the positive indirect effects of OI on employee PSRB, such that the indirect effect through EMOCB is stronger when LMX quality is high and weaker when LMX is low.

## Methods and Results

### Overview of Studies

The current study consists of two field surveys in which data was collected from employees working in Pakistan in three time-lags through a self-administered questionnaire. The first study tested the mediating role of EMOCB between OI and PE and the moderating role of LMX between OI and EMOCB. Data for the first study was collected from employees working in the service sector, particularly universities, banks, and telecommunication organizations. The service sector consists of businesses that provide a wide range of services to the customers ranging from physical services to transfer of knowledge. We used a constructive replication approach (Lykken, 1968) by using diverse datasets to enhance the external and internal validity of our findings. The second study was more extensive as it (a) validated the results of study 1 in the hospitality industry which also comes under service industry but involves generous reception of customers and is fast growing in Pakistan. Also it is less studied particularly in the context of OI, (b) tested an additional dependent variable that is pro-organizational namely UPOB (c) investigated a form of deviant pro-social behavior as an outcome variable namely PSRB (d) tested the mediating role of EMOCB between OI and self-serving behavior namely PE, organizational serving behavior namely UPOB and social serving behavior namely PSRB and (e) tested the moderating role of LMX in a different industry. Researchers believe that multiple surveys conducted in different sectors validate the results and increase the generalizability of the findings. Social sciences researchers are advised to conduct multiple studies to test the same models as it boosts confidence in the results and offers practical implications to a wider population (Harold and Holtz, 2015).

### Sample and Procedure

Before data collection, the authors personally visited the human resource department of different organizations and officially took permission for data collection from the human resource manager. The human resource managers were requested to let the research team approach the respondents at the workplace to ensure the confidentiality of the respondents. After receiving permission, the researchers approached the employees and told them about the purpose of the study. Questionnaires were distributed to those employees who volunteered to participate in the study. To get maximum responses at all three-time lags, the respondents were told that those respondents who will submit responses at all three-time lags will be given the chance to participate in the lucky draw. The researchers personally visited the organizations at all three time lags for data collection. At the end of the data collection process, two winners were announced (one for study 1 and other for study 2). The winners received 5,000 PKR. Since the total population of employees working in the service and hospitality sector is unknown, we used a non-probability purposive sampling technique. Data for both studies were collected by using a 5-point Likert scale ranging from 1 = strongly disagree and 5 = strongly agree. The current study adopted well-established scales for all variables in both surveys. Questionnaires were distributed in English language as it is the official language of Pakistan and is commonly used in Pakistan work settings. Other researchers also collected data from Pakistani samples in the English language and did not face any language-related issues (Irshad et al., 2020; Sarwar et al., 2020).

G^∗^Power (version 3.1.9.4) was employed to check the adequacy of sample sizes. The medium effect size (0.15), an alpha level (0.95) with high power (0.95) was set in the input parameters that is well above the minimum requirement (0.80) recommend by Cohen’s (1992), the number of predictors were set to 3 due to maximum number of arrows to the mediating variable EMOCB (i.e., OI, LMX, and OI^∗^LMX; Memon et al., 2020). The minimum sample size required for our study with high power of 0.95 is 119. Thus, our sample size 356 for study 1 and 259 for study 2 is adequate to test the hypothesized model.

### Analytical Strategy

Hayes’ PROCESS macro developed for SPSS was used to test the hypotheses in both studies. Our model formally included mediation, moderation, and moderated mediation relationships. Model 4 of the PROCESS macro was used to test for mediation, Model 1 for the moderation analysis, and Model 7 for the overall moderated mediation model. The same strategies have been used by past researchers to analyze similar models (Yoo and Lee, 2019; Lan et al., 2020).

### Study 1

For Study 1, 500 printed questionnaires were distributed. Employees were asked about their level of OI and their exchange relationship with their supervisor along with their demographic information. 436 responses were received back. At Time 2, the 436 respondents from Time 1 were traced through their assigned key and asked to fill out a survey containing questions regarding their EMOCB. 393 responses were obtained at Time 2. At Time 3, employees who provided data in the first two waves were contacted and asked to answer questions regarding their PE. 363 out of 393 employees responded to the third wave of the study. After matching the keys for all three time waves and eliminating incorrectly filled-out questionnaires, 356 completed questionnaires were considered in the final analysis.

The sample consisted of 64% males and 36% females. 19.7% of respondents were less than 25 years of age, 46% fell within the age bracket of 26–33 years of age, 32.3% were between 34 and 49 years of age, and 2% were 50 years of age or older. 21.7% of respondents had below a bachelor’s degree, 57% had a bachelor’s degree, and 21.3% had master’s degree or above. 71.3% of respondents had one to 5 years of job experience, 17.4% of respondents had 6–10 years of experience, and 11.3% of respondents had more than 10 years of job experience.

### Measures for Study 1

Employees’ gender, age, education, and experience were added as control variables in the present study. Details of study variables is given below:

#### Organizational Identification

A six-item scale developed by Mael and Ashforth (1992) was adopted to measure employees’ OI. A sample item is: “When someone criticizes my organization, it feels like a personal insult.” The alpha reliability of this scale was 0.80 in the current study.

#### Externally Motivated Organizational Citizenship Behavior

Employees’ EMOCB was measured with a five-item scale by Yam et al. (2017). Sample items are: “I have the skills that are needed to make this change work” and “I engage in organizational citizenship behavior because others will reward me.” The alpha reliability value for this scale was 0.77.

#### Leader–Member Exchange

The seven-item scale developed by Graen and Uhl-Bien (1995) was adopted in this study. A sample item reads: “Regardless of how much formal authority he has built into his position, what are the chances that your leader would use his power to help you solve problems in your work?” This scale’s reliability in the current study is 0.79.

#### Psychological Entitlement

The nine-item measure developed by Campbell et al. (2004) was adopted to measure sense of psychological entitlement. A sample item read: “I feel entitled to more of everything.” Cronbach alpha for this scale was 0.81. One item of this scale was context related. Its statement was: “if I were on the Titanic, I would deserve to be on the first lifeboat.” The researchers personally administered the data collection process and explained the context behind this statement to the respondents. None of the respondents faced any issue in this statement or any other statement. Other researchers have also used the same scale for collecting data on psychological entitlement in a similar context and did not face any language-related or context related issues (Naseer et al., 2019).

### Preliminary Analysis

According to the ANOVA results, gender was not associated with significant variance in the dependent variable. Age, education and job experience were associated with significant variance in psychological entitlement (*F* = 4.54, *p* < 0.01), (*F* = 19.00, *p* < 0.01), and (*F* = 4.63, *p* < 0.01), respectively.

### Confirmatory Factor Analysis

Before testing the hypothesized relationships, confirmatory factor analysis was conducted to test the model’s fit to the collected data. CFA results of study1 for the four-factor model of OI, LMX, EMOCB and PE provides best fit indices (χ^2^ = 528, df = 287, χ^2^/df = 1.83, CFI = 0.92, IFI = 0.92, TLI = 0.91, RMESA = 0.05) than combining all into a single factor (χ^2^ = 1317, df = 293, χ^2^/df = 4.50, CFI = 0.66, IFI = 0.66, TLI = 0.63, RMESA = 0.10). These results prove that the collected data is more fitted to four factors than one factor.

### Descriptive Statistics and Correlations

Table 1 shows the means, standard deviations, reliability coefficients, and correlation analysis for the variables under study. The results show that all variables are significantly correlated with each other. The correlation between OI and LMX was found positive and significant (*r* = 0.49, *p* < 0.01), which might cause the problem of multi-collinearity. To address the issue of multi-collinearity, a two-factor confirmatory analysis model fitness results were compared with one factor. Two factor model for OI and LMX yielded better fit indices (χ^2^ = 161.64, df = 64, χ2/df = 2.52, CFI = 0.92, IFI = 0.92, TLI = 0.90, RMESA = 0.06), than one factor model (χ^2^ = 363.98, df = 65, χ^2^/df = 5.60, *p* < 0.001; CFI = 0.75, IFI = 0.75, TLI = 0.70, RMESA = 0.11). It proves that respondents have provide the data for OI and LMX separately rather than considering them one factor. Furthermore, collinearity diagnostic test was employed and variance inflation factor (VIF) value was 1.32 and tolerance value 0.75 which fall within the threshold value limit for high multi-collinearity, i.e., VIF > 0.10 and Tolerance < 0.10 (Hair et al., 2014). Therefore multi-collinearity is not a problem in our study.

**TABLE 1:** Study 1: descriptive statistics, correlations, and reliabilities.

	**Variables**	**Mean**	***SD***	**1**	**2**	**3**	**4**	**5**	**6**	**7**
(1)	OI	3.31	0.80	**(0.80)**						
(2)	EMOCB	3.27	0.82	0.36**	**(0.77)**					
(3)	PE	3.45	0.78	0.46**	0.51**	**(0.81)**				
(4)	LMX	3.24	0.83	0.49**	0.39**	0.24**	**(0.79)**			
(5)	Age	–	–	−0.07	−0.03	−0.02	−0.05			
(6)	Education	–	–	0.11*	0.16**	0.28**	0.08	0.44**		
(7)	Experience	–	–	0.18*	0.11*	0.21**	−0.01	0.63**	0.13*	

N = 356, *p < 0.05, **p < 0.01 (2-tailed). Cronbach Alpha Reliabilities are given in bold and parenthesis. OI, organizational identification; EMOCB, externally motivated organizational citizenship behavior; PE, psychological entitlement; LMX, leader–member exchange.

### Hypothesis Testing

Study 1 bootstrapped results for the direct and indirect effects are presented in Table 2. The results show that OI leads to an increase in PE (β = 0.27, *p* < 0.001). Hence a result of Study 1 provides support to acceptance of H1. Table 2 illustrates that OI has a significant indirect effect on PE *via* EMOCB (*indirect effect* = 0.12), as the lower and upper limits (0.07, 0.19) of the 95% confidence intervals do not include zero. Hence, the results of Study 1 also support H4.

**TABLE 2:** Study 1: bootstrapping results for direct and indirect effects.

	**Paths**	**Coefficient**	***S.E***	***t***		**Decision**
**Study 1**						
**Control variable**						
	Age → PE	−0.11*	0.05	−2.25		
	Education → PE	0.21**	0.04	4.85		
	Experience → PE	0.19**	0.06	3.23		
**H1**	OI → PE	0.27**	0.04	6.21		Accepted
	OI → EMOCB	0.37**	0.05	7.34		
	EMOCB → PE	0.33**	0.04	7.84		

**Indirect effect (bias corrected confidence interval method)**						

		**Indirect effect**	***S.E***	**LL95% CI**	**UL 95% CI**	

**H4**	OI → EMOCB → PE	0.12	0.03	0.07	0.19	Accepted

N = 356, *p < 0.05, **p < 0.01; Bootstrap sample size = 2000. LL, lower limit; CI, confidence interval; UL, upper limit. OI, organizational identification; EMOCB, externally motivated organizational citizenship behavior; PE, psychological entitlement.

**FIGURE 2:**
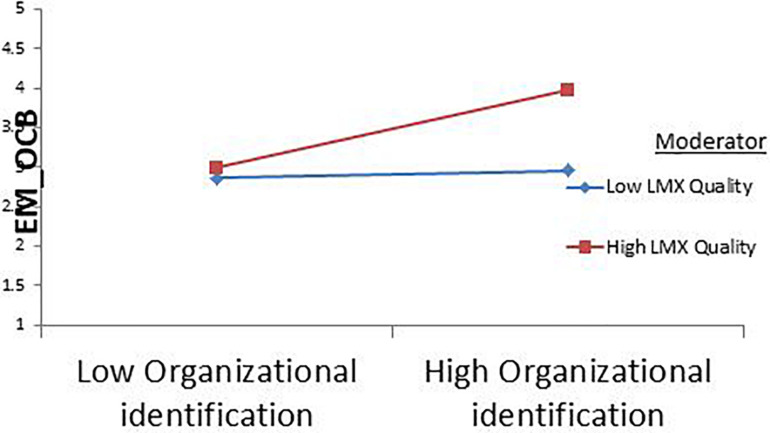
Study 1 moderation graph.

In Table 3, the bootstrap results at a 95% confidence interval indicate that interaction effect of OI and high-quality LMX on EMOCB is significant (β = 0.21, *p* < 0.001). The change in *R* squared due to the interaction effect is (Δ*R*^2^ = 0.04, *P* < 0.01). The simple slopes test also indicated that the relationship between OI and EMOCB is stronger and significant at +1 standard deviation above the mean value (β = 0.44, *CI* [0.29, 0.59]) than at −1 standard deviation below the mean value of the moderator LMX quality (β = 0.09, *CI* [−0.03, 0.22]). Furthermore, the moderation graph for Study 1 showed that high-quality LMX strengthens the relationship between OI and EMOCB compared to low-quality LMX. Hence, H7 is supported. Figure 2 shows the moderation graph for study 1.

**TABLE 3:** Study 1: moderation analysis.

		**β**	***S.E***	**Δ*R*^2^**	**Decision**
	Constant	3.20**	0.04		
	OI → EMOCB	0.27**	0.05		
	LMX → EMOCB	0.29**	0.06		
**H7**	OI × LMX → EMOCB	0.21**	0.05	0.04	Accepted

Conditional effects of (LMX) at *M* ± 1 *S.D.* (slope test)	**Effect**	***S.E***	**LL95 % CI**	**UL95 % CI**	

LMX Low −1 S.D. (−0.83)	0.09	0.06	−0.03	0.22	
LMX Medium *M* (0.00)	0.27	0.05	0.16	0.38	
LMX High + 1 SD (0.83)	0.44	0.07	0.29	0.59	

N = 356, **p < 0.01. *OI, organizational identification; EMOCB, externally motivated organizational citizenship behavior; LMX, leader–member exchange.*

The moderated mediation results for Study 1 are presented in Table 4, which indicates that the conditional indirect effect of OI on PE through EMOCB becomes stronger and significant at +1 standard deviation above the mean of LMX quality (β = 0.15, *CI* [0.08, 0.23]) than at −1 standard deviation below the mean of LMX quality (β = 0.02, *CI* [−0.02, 0.08]). Hence, H8 is fully supported by the results of Study 1.

**TABLE 4:** Study 1: moderated mediation.

**H8**	**Conditional indirect effects of OI at** **values moderator LMX mean and** **±1 SD on PE through mediator** **EMOCB**	**Dependent variable** **Psychological entitlement**				**Decision**
		**Bootstrapping**		**Bias 95% *CI***		
		**Effect**	***SE***	**LL**	**UL**	
LMX low −1 *SD* (−0.83)		0.02	0.03	−0.02	0.08	
LMX medium *M* (0.00)		0.09	0.03	0.04	0.14	
LMX high +1 *SD* (0.83)		0.15	0.04	0.08	0.23	Accepted

*N = 356, *LL, lower limit; CI, confidence interval; UL, upper limit. OI, organizational identification; EMOCB, externally motivated organizational citizenship behavior; PE, psychological entitlement; LMX, leader–member exchange*.*

### Study 2

For the replication and generalizability of results, we conducted another time-lagged study in a different sector (i.e., hospitality). Two additional outcome variables (i.e., UPOB and PSRB) of organizational identification through EMOCB were also added to extend the model of Study 1.

### Sample and Procedure

Four hundred questionnaires were distributed among employees in the Pakistani hospitality sector. Similar to Study 1, employees were asked to report their demographics, level of OI, and quality of LMX relationship at T1. At T2, the same employees were asked to report their EMOCB. At T3, employees were asked to report their PE, UPOB, and PSRB. The same procedure as in Study 1 was followed to track employees across the different waves. We distributed 400 questionnaires at T1 and received 343 surveys back. These 343 respondents were approached at T2 2 weeks later. Of these 343 respondents, 312 returned the questionnaires at T2. These 312 respondents were then invited to participate at T3, and we received 272 questionnaires back. After removing incomplete and mismatched responses, 259 complete questionnaires were obtained at the end of T3 and considered in the analysis.

The total response rate was 64.8%. Among these 259 complete responses, 166 were male and 93 were female. 31.7% were between 18 and 25 years of age, 45.3% from 26 to 33 years of age, and the remaining 23% have above 34 years of age. In terms of education, 5% of respondents had a 10th grade completion certificate (‘matric’ in Pakistan), 17.3% had a high school diploma (‘FSC’), 53.7% have a bachelor’s degree, and 24% had a master’s degree or higher. 71.8% of respondents had less than 5 years of experience, 16.2% had between 6 and 10 years of experience, and 12% had more than 10 years of experience.

### Measures for Study 2

We measured OI, EMOCB, PE, and LMX quality using the same scales as in Study 1. Alpha reliabilities for the scales in Study 2 were 0.84 for OI, 0.79 for EMOCB, 0.89 for PE and 0.85 for LMX quality, respectively.

#### Unethical Pro-organizational Behavior

The six-item scale used to measure UPOB was adopted from Umphress et al. (2010). A sample item is: “If needed, I would conceal information from the public that could be damaging to my organization.” The alpha reliability of the six-item scale was 0.85.

#### Pro-social Rule-Breaking

The 13-item scale developed by Dahling et al. (2012) comprised questions regarding rule-breaking for efficiency, helping customers and coworkers. A sample item is: “I break organizational rules or policies to do my job more efficiently.” The alpha reliability for the thirteen-item scale was 0.90.

### Preliminary Analysis

An analysis of variance (ANOVA) was performed to check the control variables impact on dependent variables. Significant variance in PE was found due to education (*F* = 11.23, *p* < 0.01), and experience (*F* = 2.78, *p* < 0.05).

### Confirmatory Factor Analysis

For study 2, CFA results of the six-factor model of OI, LMX, EMOCB, PE, UPOB, and PSRB provides best fit indices (χ^2^ = 1420, df = 971, χ^2^/df = 1.46, CFI = 0.91, IFI = 0.91, TLI = 0.90, RMESA = 0.04) than combining all into a single factor (χ^2^ = 3851, df = 986, χ^2^/df = 3.90, CFI = 0.43, IFI = 0.40, TLI = 0.44, RMESA = 0.11). These results prove that the collected data for study 2 is best fitted to six factors than one factor.

### Descriptive Statistics and Correlations

Means, standard deviations, reliabilities score and inter-correlations among variables in Study 2 are presented in Table 5. The results show that all variables are significantly correlated with each other.

**TABLE 5:** Study 2: descriptive statistics, correlations, and reliabilities.

	**Variables**	**Mean**	***SD***	**1**	**2**	**3**	**4**	**5**	**6**	**7**	**8**	**9**
(1)	OI	3.16	0.88	**(0.84)**								
(2)	EMOCB	3.30	0.85	0.31**	**(0.79)**							
(3)	PE	3.40	0.85	0.26**	0.48**	**(0.89)**						
(4)	UPOB	3.42	0.87	0.22**	0.23**	0.17**	**(0.85)**					
(5)	PSRB	3.75	0.78	0.24**	0.25**	0.32**	0.24**	**(0.90)**				
(6)	LMX	3.25	0.93	0.24**	0.27**	0.13*	0.23**	0.12*	**(0.85)**			
(7)	Age	–	–	0.05	0.01	0.11	0.02	0.08	0.08			
(8)	Education	–	–	0.02	0.18**	0.30**	−0.02	0.06	0.09	0.39**		
(9)	Experience	–	–	0.23**	0.07	0.18**	0.06	0.08	−0.04	0.66**	0.14*	

N = 259, *p < 0.05, **p < 0.01 (2-tailed). Cronbach alpha reliabilities are given in bold and parenthesis. OI, organizational identification; EMOCB, externally motivated organizational citizenship behavior; PE, psychological entitlement; UPOB, unethical pro-organizational behavior; PSRB, pro-social rule breaking; LMX, leader–member exchange.

### Hypothesis Testing

Regression results for the direct and indirect effects examined in Study 2 are presented at the Table 6. The results of Study 2 are in line with those of Study 1, thus replicating the first study’s overall contribution and demonstrating its generalizability. OI was found to be significantly associated with PE (β = 0.15, *p* < 0.001), as well as with the additional outcome variables UPOB (β = 0.17, *p* < 0.001) and PSRB (β = 0.16, *p* < 0.001). Thus, the results of Study 2 support H1, H2 and H3.

**TABLE 6:** Study 2: bootstrapping results for direct and indirect effects.

		**Paths**		**Coefficient**	***S.E***	***t***	**Decision**
**Control variable**							
		Education → PE		0.20**	0.05	3.90	
		Experience → PE		0.12*	0.06	1.93	
		OI → EMOCB		0.30**	0.05	5.21	
		EMOCB → PE		0.40**	0.06	7.20	
**H1**		OI → PE		0.15**	0.06	2.93	Accepted
**H2**		OI → UPOB		0.17**	0.06	2.69	Accepted
		EMOCB → UPOB		0.18**	0.06	2.77	
**H3**		OI → PSRB		0.16**	0.06	2.87	Accepted
		EMOCB → PSRB		0.18**	0.06	3.17	

**Indirect effect (bias corrected confidence interval method)**							

			**Indirect effect**	***S.E***	**LL95% CI**	**UL 95% CI**	

**H4**	OI → EMOCB → PE		0.12	0.03	0.06	0.19	Accepted
**H5**	OI → EMOCB → UPOB		0.05	0.02	0.02	0.11	Accepted
**H6**	OI → EMOCB → PSRB		0.05	0.02	0.02	0.11	Accepted

N = 259, *p < 0.05, **p < 0.01; Bootstrap sample size = 2,000. LL, lower limit; CI, confidence interval; UL, upper limit. OI, organizational identification; EMOCB, externally motivated organizational citizenship behavior; PE, psychological entitlement; UPOB, unethical pro-organizational behavior; PSRB, pro-social rule breaking.

The indirect effects for Study 2 are presented at the bottom of Table 6. OI has a significant indirect effect (*indirect effect* = 0.12) on PE via EMOCB, as the confidence interval did not include zero (0.06, 0.19). Thus, H4 is also verified by the results of Study 2. OI also had a significant indirect effect (*indirect effect* = 0.05) on UPOB, as the confidence interval did not include zero (0.02, 0.11). Additionally, OI had a significant indirect effect (*indirect effect* = 0.05) on PSRB, as the confidence interval did not include zero (0.02, 0.11). Hence, Study 2 results provide support for H4, H5, and H6.

**FIGURE 3:**
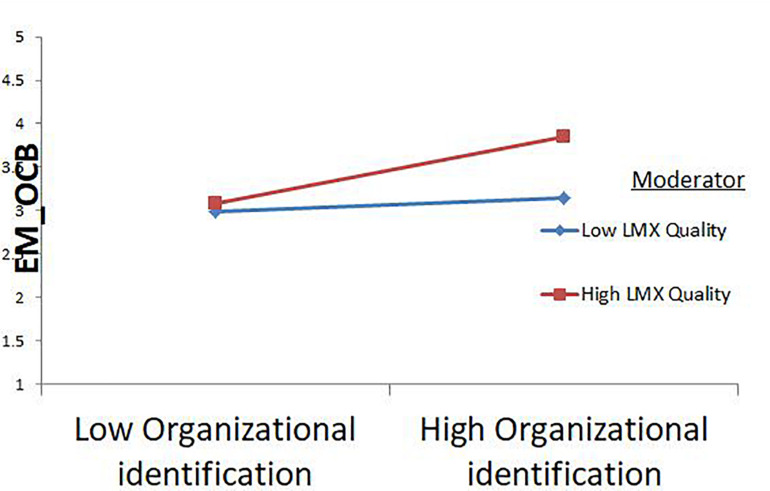
Study 2 moderation graph.

The results of Study 2, in Table 7 indicate that high-quality LMX strengthened the positive relationship between OI and EMOCB, as the interaction effect was significant (β = 0.15, *p* < 0.001). The change in *R* squared due to the moderating effect was (Δ*R^2^* = 0.02, *p* < 0.01). The slope test for high-quality LMX is positive and significant (β = 0.37, *CI* [0.23, 0.52]), while the slope test results for low-quality LMX is not significant (β = 0.10, *CI* [−0.05, 0.25]). The moderation graph for Study 2 also demonstrates that high-quality LMX enhances the relationship between OI and EMOCB. Hence study also justifies result of H7. Figure 3 shows the moderation graph for study 2.

**TABLE 7:** Study 2: moderation analysis.

		**β**	***S.E***	**Δ*R*^2^**	**Decision**
	Constant	3.27**	0.05		
	OI → EMOCB	0.23**	0.05		
	LMX → EMOCB	0.20**	0.05		
**H7**	OI × LMX → EMOCB	0.15**	0.05	0.02	Accepted

Conditional effects of (LMX) at *M* ± 1 *S.D.* (slope test)	**Effect**	***S.E***	**LL95 % CI**	**UL95 % CI**	

LMX low −1 *SD* (−0.92)	0.10	0.08	−0.05	0.25	
LMX medium *M* (0.00)	0.23	0.05	0.12	0.35	
LMX high +1 *SD* (0.92)	0.37	0.07	0.23	0.52	

N = 259; **p < 0.01. *OI, organizational identification; EMOCB, externally motivated organizational citizenship behavior; LMX, leader–member exchange*.

Table 8 presents the results of the moderated mediation model. OI had a significant indirect effect through EMOCB on PE (β = 0.12, *CI* [0.05, 0.22), UPOB (β = 0.06, *CI* [0.02, 0.13), and PSRB (β = 0.07, *CI* [0.02, 0.13]) in the case of high-quality LMX. Conversely, the indirect effect of OI via EMOCB on PE (β = 0.03, *CI* [−0.03, 0.09]), UPOB (β = 0.02, *CI* [−0.01, 0.07]), and PSRB (β = 0.02, *CI* [−0.01, 0.07]) becomes insignificant and low when LMX is of low quality. These results provide support for H8, H9, and H10.

**TABLE 8:** Study 2: moderated mediation.

**Conditional indirect** **effects of OI at values moderator LMX mean** **and ±1 *S.D*. on DV’s** **through mediator** **EMOCB**	**Dependent variables**											
	**H8: PE**				**H9: UPOB**				**H10: PSRB**			
	**Bootstrapping**		**Bias 95% *CI***		**Bootstrapping**		**Bias 95% *CI***		**Bootstrapping**		**Bias 95% *CI***	
	**Effect**	***SE***	**LL**	**UL**	**Effect**	***SE***	**LL**	**UL**	**Effect**	***SE***	**LL**	**UL**
LMX low −1 *SD* (−0.92)	0.03	0.03	−0.03	0.09	0.02	0.02	−0.01	0.07	0.02	0.09	−0.01	0.07
LMX medium *M* (0.00)	0.08	0.03	0.03	0.14	0.04	0.02	0.01	0.09	0.04	0.09	0.01	0.09
LMX high +1 *SD* (0.92)	0.12	0.04	0.05	0.22	0.06	0.03	0.02	0.13	0.07	0.03	0.02	0.13
**Decision**	Accepted				Accepted				Accepted			

*N = 259, LL, lower limit; CI, confidence interval; UL, upper limit. *OI, organizational identification; EMOCB, externally motivated organizational citizenship behavior; PE, psychological entitlement; UPOB, unethical pro-organizational Behavior; PSRB, pro-social rule breaking; LMX, leader–member exchange.**

## Discussion

Since its initial conceptualization by Ashforth and Mael (1989), OI has been perceived as a positive phenomenon offering a wide range of benefits for employees as well as organizations. However, this “optimistic view” has started to dim after repeated criticism from organizational behavior scholars (Naseer et al., 2019). However, existing studies have not fully revealed the underlying mechanisms and boundary conditions contributing to the impact of OI on adverse outcomes (Brown, 2017; Naseer et al., 2019). Another significant gap in OI research is the lack of studies on its dark side.

The current study contributes to this relatively understudied field by examining PE, UPOB, and PSRB as the negative outcomes of OI through the intervening mechanism of EMOCB. Moreover, by studying the conditional effect of LMX on the association between organizational identification and EMOCB, the current study illustrated the significance of situational factors in understanding the OI-outcome relationship. The results supported the hypothesized moderated mediation model of a strong indirect relationship between OI and employee outcomes – specifically PE, UPOB, and PSRB through EMOCB in the case of a high-quality LMX relationship. These results not only respond to calls to study the negatives outcomes of organizational identification but also open new research avenues for future studies to explore. These results have endorsed the repeated claims that organizational identification can prove to be detrimental for the organizations. This study is timely as it has identified three important negative outcomes of organizational identification and the underlying mechanism that cause these outcomes. Further, it has highlighted the role of LMX in strengthening the relationship between OI and negative employee outcomes.

The results of the current study validate the assumptions of social identity theory in the service industry (i.e., Universities, Banks, and Telecommunication Organizations) and hospitality industry both. This theory talks about social identification with the group and its consequences. According to this theory, membership of a certain group gives employees a feeling of pride, and these members of a particular group also expect to experience beneficial outcomes from acting in accordance with group norms (Tajfel and Turner, 1986). The results also supported this association by providing support to the OI-PE relationship. This theory further states that members of a group are willing to engage in behaviors that benefit the group and its stakeholders. The current study validated this assumption by providing support for the impact of OI on UPOB and PORB. The core assumption of social identity theory is that employees high in OI feel obligated to engage in behaviors that are ingrained in the organizational culture and expected from organizational members. One such behavior frequently expected by contemporary organizations is EMOCB. Since employees with higher levels of OI are obligated to fulfill organizational norms due to their in-group assimilation, they are more likely to display EMOCB due to group expectations (Tajfel and Turner, 1985). The results support this assumption by supporting the mediating hypotheses. Additionally, this theory posits that those individuals who feel identified with the group and are close to the people who are in power in the group are more likely to accept group norms. The significant results of moderation hypothesis supported this assumption.

The current study has several strengths. First, it adds to the existing body of knowledge on the negative side of OI by proposing PE, UPOB, and PSRB as outcomes. Second, it untangles the dark side of EMOCB by studying it as an underlying mechanism in the relationship between OI and negative outcomes. Third, it studies the conditional effect of a situational factor, LMX, on the OI-outcome relationship. Fourth, it validates research on organizational identification by studying underexplored outcomes as well as a unique underlying mechanism and boundary condition in the Pakistani context, which is characterized by high power distance and collectivism.

In addition to these important theoretical contributions, the study has various methodological strengths. First, the current study tested the outcomes of OI on two different samples by conducting multi-wave surveys. Study 1 tested a single adverse outcome of OI (i.e., PE), whereas Study 2 not only replicated and generalized the results of Study 1 but also studied two additional consequences (i.e., UPOB and PSRB). Other strengths of this study include its time-lagged research design and use of the bootstrapping method.

### Limitations and Future Research Directions

Despite offering substantial theoretical and methodological contributions, the current study also has a few limitations. First, this study used time-lagged data. Second, the sample consisted only of employees working in the service and hospitality sectors, which decreases the generalizability of the findings.

Given these limitations, future researchers may replicate our study by utilizing a longitudinal research design in a relatively different context. It is also recommended to study this model in the manufacturing sector. Moreover, in light of the significance of research on OI and its adverse outcomes, researchers are suggested to identify other mediating and moderating mechanisms to further enhance our understanding of the processes and boundary conditions that cause adverse outcomes of OI. It would also be fruitful to study the antecedents of OI. For instance, researchers could identify leadership styles which can increase organizational identification among employees to optimal level. Additionally, the conditional effect of individual differences, such as the Big Five personality traits and the ‘dark triad’, could be studied in the future.

### Practical Implications

Our study offers valuable insights for managers. First, the positive association between OI and PE indicates that a high level of OI is detrimental for organizations. Hence, managers should be proactive in identifying and addressing employees who exhibit a high level of OI to stop them from becoming entitled. One way of doing so is to institute a transparent reward system based on performance so that employees are clear that they will only be rewarded if they show good performance. Organizations must also discourage employees from demanding preferential treatment by creating a strict policy that clearly shows that no extra favors will be given to any employee. Second, the positive association between OI and UPOB should encourage managers to develop a strict organizational ethical code of conduct to discourage employees from engaging in unethical behaviors, even when they are pro-organizational. Managers should act as role models. They must refrain from engaging in unethical behaviors even if they are beneficial for the organization. Those employees who are engaged in these behaviors should be given oral or written warning to discourage them from engaging in these behaviors in the future. Third, managers should be rigid when it comes to organizational rules. Strict policies should be in place to discourage employees from breaking the rules even if it is beneficial for the organization and its stakeholders. Those employees who break the rules must be given warning to stop employees from developing the culture of rule breaking. One way of doing so is linking rule following with job performance and rewards. Those employees should be given monetary or non-monetary rewards who refrain from engaging in unethical pro-organizational behaviors and rule breaking. Forth, the adverse outcomes of EMOCB indicate that employees don’t like to be forced to engage in OCB; hence, managers should not obligate employees to show OCB. Fifth, employees who are part of the leader’s in-group should not be given any indirect signals to do more than what is required from their job, as external pressure to engage in extra-role behavior can prove to be dangerous for the organization, as it can promote negative behaviors.

### Conclusion

There has been a paradigm shift from positive side of OI to its dark side. The current study has contributed toward this underexplored side of OI by investigating its (a) negative employee outcomes, (b) underlying mechanism, and (c) boundary condition. These results offer important insights to organizational behavior researchers as well as practitioners regarding the negative side of OI and its adverse outcomes.

## Data Availability Statement

The raw data supporting the conclusions of this article will be made available by the authors, without undue reservation.

## Ethics Statement

The studies involving human participants were reviewed and approved by FMS Research Ethics Board, Capital University of Science and Technology, Islamabad, Pakistan. The patients/participants provided their written informed consent to participate in this study.

## Author Contributions

All authors listed have made a substantial, direct and intellectual contribution to the work, and approved it for publication.

## Conflict of Interest

The authors declare that the research was conducted in the absence of any commercial or financial relationships that could be construed as a potential conflict of interest.
